# Editorial: Whole-body electromyostimulation: A training technology to improve health and performance in humans? volume II

**DOI:** 10.3389/fphys.2022.972011

**Published:** 2022-08-05

**Authors:** Wolfgang Kemmler, Heinz Kleinöder, Michael Fröhlich

**Affiliations:** ^1^ Institute of Radiology, University Hospital Erlangen, Erlangen, Germany; ^2^ Institute of Medical Physics, Friedrich-Alexander University of Erlangen-Nürnberg, Erlangen, Germany; ^3^ Institute of Training Science and Sport Informatics, German Sport University Cologne, Cologne, Germany; ^4^ Department of Sports Science, Technische Universität Kaiserslautern, Kaiserslautern, Germany

**Keywords:** whole-body electromyostimulation, health, performance, low back pain, knee arthrosis, seniors, critically–ill patients

Launched in Germany in about 2006, whole-body electromyostimulation (WB-EMS) is a young exercise technology. Due to its increasing popularity, time-efficiency, joint-friendliness and individualized application, WB-EMS has been increasingly been the subject of scientific research. Apart from the current Research Topics, the large variety of articles published during the last 2 years has considerably increased the evidence for positive effects on relevant health and performance related parameters. Notably, there is a clear tendency towards the scientific application of WB-EMS in the area of “health” that have been mirrored in the present Research Topic. Summarizing the published articles, at least six of seven contributions addressed health-related outcomes while one study Verch et al. can be considered as a methodological trial that determined the specific effect of WB-EMS combined with walking and Nordic Walking on oxygen consumption (VO_2_) in healthy adults. Although the authors reported significant net effects on average VO_2_ (≈10%) during the 10 min treadmill test, they doubt the clinical relevance of their results. In line with similar data of longitudinal studies (e.g. Mathes et al., 2017), this result questioned (once again) the concept of superimposed WB-EMS application during endurance exercise at least in non-athletic cohorts.

In their meta-analysis Kemmler et al., on the other hand, provided strong evidence for a large effect of WB-EMS on muscle mass and strength parameters in non-athletic adult cohorts. However, although six of the twelve trials included obese participants, the authors failed to determine significant effects on body fat parameters, - still a favorite training aim in commercial WB-EMS (Rodrigues-Santana et al., 2022). Applying magnetic resonance imaging, Zink-Rückel et al. provided a deeper insight into WB-EMS effects on “muscle quality” in men. Considering fatty muscle infiltration as a relevant and reliable predictor of “muscle quality”, the authors determined significant positive effects on intramuscular adipose tissue (IMAT) and muscle tissue volume of the mid-thigh after 16 weeks of once weekly 20 min WB-EMS standard application (i.e. bipolar, 85 Hz, 350 µs, 4s impulse—4s impulse break). Apart from its scientific merits, the study also provided evidence for the feasibility, attractiveness, and effectiveness of a consistently supervised WB-EMS protocol at the participant’s location.

Another important aim of WB-EMS, the relief of back pain, can now be considered as confirmed. In line with recent studies (Weissenfels et al., 2018; Konrad et al., 2020), the authors reported significant effects of WB-EMS on low-back pain (LBP) after 12 weeks of WB-EMS and thus questioned the “negative recommendation” (i.e., “do not”) in the German Guideline on (Low) Back Pain (LBP) for all kinds of electrotherapy. In detail, the large sized comparative study of Micke et al. observed similar, clinically relevant changes of LBP after once weekly WB-EMS standard application compared with two recognized LBP exercise programs. From the patient’s point of view, this finding is a dream result - depending on time availability and personal preferences, they can choose between different training options for pain management.

Comparable to the issue of low back pain, a Korean study by Park et al. applied WB-EMS in an area (knee osteoarthrosis, OA) which would be rather located in the domain of local EMS. The rationale for whole-body application stated by the authors was its hypothesized impact on sub-clinical (low-grade) inflammation, potentially involved in the pathologic process of OA. After 8 weeks of 3x30 min/w. WB-EMS (standard) application combined with isometric exercise, the authors reported significant positive effects for knee pain, symptoms, function and activity of daily life (ADL) compared with the isometric exercise intervention. In parallel, changes in biomarkers were much more pronounced in the combined vs the isolated isometric exercise group.

In their pilot study, Bloeckl et al. confirmed the feasibility, attractiveness, safety and effectivity of WB-EMS application inter alia in frail older habitants of assisted living facilities. In detail, 8 weeks of WB-EMS standard application resulted in significant and meaningful changes in the SPPB, maximum leg and handgrip strength and reaction time that were also more favorable compared with WB-EMS-induced changes of a physically more robust cohort of comparable age. With respect to safety, pre-, 48 and 72 h post exercise creatine-kinase kinetics frequently determined during the intervention do not indicate any relevant rhabdomyolytic hazard; furthermore, no adverse effects related to the WB-EMS application were reported.

Finally, the potentially most vulnerable group, namely critically ill patients (e.g., intensive care, invasive ventilation patients), was addressed by the systematic review of Balke et al. In contrast to all the other studies of the present Research Topic, however, all kinds of EMS application involving WB-EMS were included. In summary, the authors found still inconclusive evidence for positive effects on functional independence, muscle function or weaning outcomes. Nevertheless, Balke et al. recommended the application of appropriate and dedicated EMS protocols on top of existing rehabilitation programs.

Summing up the present Research Topic, WB-EMS predominately applied as resistance type exercise with moderate-high intensity, intermitted impulse, short duration and low training frequency can be considered a feasible and efficient training technology for addressing important health-related outcomes widely independently of the functional status of the participant/patient. Of crucial relevance, all the trials involved consistently supervised and instructed WB-EMS with quite a low supervision ratio, an aspect that ensures both safety and efficiency, at least when applied by qualified and licensed trainers. The latter aspect has now been specified by a mandatory German standard, the NISV (“applications of non-ionizing radiation to humans” ordinance). However, new business models tend to focus on complete or largely non-supervised WB-EMS application at home. It might be a waste of time to once again stress the importance of close supervision by an experienced and attentive instructor for effective and safe WB-EMS application (Kemmler et al., 2020). Common sense alone suggests that excessive non-supervised private WB-EMS application will significantly increase the prevalence of adverse effects. Considering that according to the German radiation protection committee WB-EMS is a handbreadth away from being restricted to medical application, a further regulation by official authorities will considerably change the WB-EMS scenery and might limit WB-EMS to higher income groups.
